# A Case of Identity: *HOX* Genes in Normal and Cancer Stem Cells

**DOI:** 10.3390/cancers11040512

**Published:** 2019-04-10

**Authors:** Jessica Smith, Ahmad Zyoud, Cinzia Allegrucci

**Affiliations:** 1 SVMS, Faculty of Medicine and Health Sciences, University of Nottingham, Sutton Bonington Campus, Loughborough LE12 5RD, UK; stxjt15@exmail.nottingham.ac.uk (J.S.); svxaz@exmail.nottingham.ac.uk (A.Z.); 2 Nottingham Breast Cancer Research Centre and Centre for Cancer Sciences, University of Nottingham, Centre for Biomolecular Sciences, Nottingham NG7 2RD, UK

**Keywords:** *HOX* genes, stem cells, cancer stem cells, self-renewal, differentiation, targeted therapy

## Abstract

Stem cells are undifferentiated cells that have the unique ability to self-renew and differentiate into many different cell types. Their function is controlled by core gene networks whose misregulation can result in aberrant stem cell function and defects of regeneration or neoplasia. *HOX* genes are master regulators of cell identity and cell fate during embryonic development. They play a crucial role in embryonic stem cell differentiation into specific lineages and their expression is maintained in adult stem cells along differentiation hierarchies. Aberrant *HOX* gene expression is found in several cancers where they can function as either oncogenes by sustaining cell proliferation or tumor-suppressor genes by controlling cell differentiation. Emerging evidence shows that abnormal expression of *HOX* genes is involved in the transformation of adult stem cells into cancer stem cells. Cancer stem cells have been identified in most malignancies and proved to be responsible for cancer initiation, recurrence, and metastasis. In this review, we consider the role of *HOX* genes in normal and cancer stem cells and discuss how the modulation of *HOX* gene function could lead to the development of novel therapeutic strategies that target cancer stem cells to halt tumor initiation, progression, and resistance to treatment.

## 1. *HOX* Genes Are Master Regulators of Embryonic Development

*HOX* genes encode evolutionarily conserved transcription factors that are expressed under temporal and spatial control to establish patterning and morphogenesis in the vertebrate embryo. They play a critical role during development, and either loss or gain of function of *HOX* genes can lead to homeotic transformation and abnormal formation of body structures [1]. In humans and mice, *HOX* genes are organized in four paralogous gene clusters (A, B, C, D) coding for 39 genes. Their expression is tightly controlled by their clustered organization so that their activation in the posterior part of the primitive streak occurs by a process of temporal and spatial collinearity depending on their location within the cluster. The position of *HOX* genes within the cluster corresponds to their positional expression along the anterior-posterior axis, with expression following the direction from the 3’ side (anterior) to the 5’ (posterior). Their temporal expression is also coordinated so that *HOX* genes positioned 3’ in the cluster are expressed earlier than those at the 5’ [2]. In addition, a phenomenon of posterior dominance can be observed whereby posterior genes are dominant with respect to more anterior genes when expressed in adjacent areas [3]. This coordinated expression is regulated by key developmental signals within the embryo, including Wnt ligands, retinoic acid (RA), and fibroblast growth factor (FGF) [4,5]. Important upstream regulators of *HOX* gene transcription are *CDX* genes. These are direct targets of Wnt, RA, and FGF and therefore integrate signaling pathway networks to regulate *HOX* gene expression [4].

Different structural and epigenetic parameters have been described contributing to the collinearity of *HOX* gene expression. These include their chromosomal cluster organization, *cis*-regulatory elements (enhancers and lncRNA), histone modifications, chromosome boundaries, and 3D chromatin conformation [6]. During development, the regulation of *HOX* genes is achieved by methylation of Histone H3 residues by Trithorax (TrxG) and Polycomb (PcG) group proteins, which establish the active H3K4me3 and repressive H3K27me3 marks, respectively. These epigenetic modifiers create bivalent domains and a poised state in the pre-gastrulating embryo by transcriptional control of developmental genes. In the bivalent state, H3K27me3 is dominant over the H3K4me3 mark, with genes controlled by these marks being repressed as default [7]. The removal of H3K27me3 by histone demethylases then allows the prompt expression of patterning and lineage specifier *HOX* genes following a temporal and special collinearity [8,9]. At gastrulation, trimethylation of H3K27 mediated by the Polycomb Repressive Complex 2 (PRC2) and recruitment of the Polycomb Repressive Complex 1 (PRC1) induce chromatin condensation to maintain a permanent silencing of the *HOX* genes in a lineage-specific manner [9,10]. The silencing of non-lineage tissue specific genes can then be further reinforced by DNA methylation, a stable and heritable epigenetic mark [11,12]. Therefore, during cell fate determination the chromosomal domains marked by active H3K4 or silent H3K27 methylation are mutually exclusive and are programmed to establish the *HOX* code and epigenetic memory of differentiated cells [13]. Together with the epigenetic regulation mediated by PcG and TrxG proteins, intergenic transcription of miRNA and long non-coding RNAs (lncRNA) regulate the expression of *HOX* genes. HOTAIR, HOTAIRM1, and HOTTIP are lncRNA located in the cluster, and they have been shown to configure the epigenetic profile of the *HOX* loci by association with PcG and TrxG proteins and to control gene expression either in *cis* or in *trans* [14]. For instance, the lncRNA HOTAIR is located in the *HOXC* cluster and required for the silencing of *HOXD* genes through interaction with the PRC2 complex and for the establishment of the repressive H3K27me3 mark [14]. On the other hand, HOTAIRM1 is a lncRNA located within the *HOXA* cluster that plays a role in regulating the expression of 3’ *HOXA* genes by preventing the accessibility of the lysine-specific demethylase 6A (UTX) and by delaying the expression of central genes in the cluster. The 5’ *HOXA* genes are instead regulated by the lncRNA HOTTIP, which is located upstream of *HOXA13* [15]. As a result of their epigenetic regulation, *HOX*-coded transcription factors can act as either transcriptional activators or repressors of target genes. These consist of factors that regulate diverse biological processes and include a network of transcription factors, signaling molecules, components of signaling pathways, and “realizator” genes that are directly involved in cell differentiation and tissue organization [16,17]. The diversity and specificity of HOX proteins is realized by their interaction with cofactors that belong to the TALE family. These include the PBC and MEINOX classes of transcription factors, with the PBC family comprising PBX proteins (PBX1-4) and the MEINOX family including MEIS (MEIS1-3) and PREP (PRE1-2) proteins. All of these transcription factors cooperate with HOX proteins by forming heteromeric complexes, [18,19] and their diverse and context-dependent interaction regulates cell fate decisions and specific cell functions during embryo patterning and morphogenesis.

## 2. *HOX* Genes and Cellular Identity

The concerted actions of HOX proteins and their cofactors are responsible not only for *HOX* gene homeotic function, but also for regulation of organogenesis. In this process, the function of *HOX* genes is to control cell proliferation, differentiation, migration, and apoptosis [16]. The resulting embryonic *HOX* gene expression profiles in each body region are then sustained in the adult so that cell and tissue characteristics are maintained according to a specific “*HOX* code” [20].

At the cellular level, the primary role of *HOX* genes is to regulate cell specification and tissue differentiation. During embryogenesis, the expression of *HOX* genes maintains positional information, and it is epigenetically inherited so that cells can maintain a memory of their identity during the lifetime of an organism. A significant demonstration of the role played by *HOX* genes in the regulation of cell identity is provided by their expression pattern in adult human fibroblasts. In these cells, *HOX* gene expression can predict their original embryonic position along the developmental axes [21,22]. For instance, the expression of *HOXA* and *HOXD* genes reflects the location along the proximal and distal axis of the limbs, whereas *HOXC* genes correlate with the anterior and posterior axis of the trunk. *HOXB* genes are instead associated with fibroblasts of non-dermal origin [22]. The same feature of positional identity is applicable to *HOX* gene expression profiles in smooth and skeletal muscle cells [23,24].

The expression of HOX proteins in different tissues, or in the same tissue at different anatomical locations, maintains correct cell identities during differentiation. Indeed, it has been demonstrated that *HOX* genes play a crucial role in stem cells both in embryonic and in adult tissues by controlling lineage specification, differentiation, and tissue maturation [25].

## 3. *HOX* Genes in Embryonic Stem Cells

*HOX* genes are not expressed before gastrulation, and they are not transcribed in embryonic stem cells (ESC) derived from the inner cell mass of a blastocyst [26]. ESC are pluripotent cells that can differentiate into all of the different cell types that comprise the body. *HOX* genes in ESC are regulated by bivalent chromatin domains so that they are poised for activation after differentiation. As a result, the coverage of the polycomb complex PRC2 in ESC is not as extensive as in differentiated cells with silenced *HOX* gene expression [27]. ESC present an active epigenetic repression of *HOX* genes in their undifferentiated state, with *HOX* gene activation being induced upon differentiation [28]. At this stage, histone demethylases remove the repressive H3K27me3 mark from *HOX* lineage specifier genes, whilst PcG proteins maintain cell identity through transcriptional repression of *HOX* genes that are specific to other lineages [29,30]. In the mouse, Wnt3 induces the gradual and collinear activation of the *HOX* gene cluster during gastrulation [31]. This mechanism is recapitulated in differentiating mouse epiblast stem cells with concomitant activation of *Cdx2* [32] and in human induced pluripotent stem cells with induction of a posterior mesoderm phenotype [33].

RA is another key inducer of *HOX* gene expression during ESC differentiation. RA treatment of ESC can significantly increase the induction of neural differentiation and upregulate the expression of *HOX* genes [34]. RA responsive elements (RARE) are found in the regulatory regions of many *HOX* genes which respond to RA signaling in a collinear fashion, with genes at 3’ responding to the signal more rapidly than those at the 5’ of the cluster [35,36]. RA receptor γ is essential for RA-induced activation of *HOX* gene transcription. Indeed, knockdown of RA receptor γ in mouse ESC impairs differentiation and reduces the expression of *Hoxa* and *Hoxb* cluster genes as well as the expression of their cofactors *Pbx1* and *Meis1* [37]. Importantly, RA/RA receptor γ signaling is required to remove PcG repressive marks from most bivalent *HOX* gene regulatory regions during ESC differentiation via recruitment of the H3K27me3 demethylase UTX-KDM6A [34,38,39]. Specific *HOX* genes are involved in the control of cell fate specification and differentiation of ESC into different lineages. For instance, inducible expression of *Hoxb1* in mouse ESC can induce the differentiation and expansion of posterior neural stem/progenitor cells [40]. On the other hand, *HOXB4* acts as a master regulator of hematopoietic differentiation in both mouse and human pluripotent stem cells [41,42,43]. *Hoxb4* can in fact regulate multiple transcription factors involved in hematopoiesis and chromatin modifiers, thus playing a role in establishing the epigenetic landscape of the developing hematopoietic stem/progenitor cells [41]. Finally, *Hox6* gene paralogues (*Hoxa6*, *Hoxb6*, *Hoxc6*) control the differentiation of mouse ESC into insulin producing pancreatic cells [44]. Therefore, the role of *HOX* genes during embryonic development is mirrored in ESC and their expression allows the control of ESC differentiation to the three germ layers.

## 4. *HOX* Genes in Adult Stem Cells

The positional identity provided by *HOX* gene expression during development and differentiation into adult tissues provides a mechanism for imposing cell identity and fate restriction. This information is maintained in adult stem cells (ASC) and along their differentiation hierarchies. This mechanism can be clearly observed in mesenchymal stem cells (MSC). These cells, isolated from different tissues including the bone marrow and adipose tissue, can differentiate to fat, bone, and cartilage. Despite their phenotypic similarity, MSC derived from different tissues present a profile of *HOX* gene expression that mirrors that of their developmental origin. The topographical specificity of *HOX* genes in MSC is also maintained after differentiation, indicating that *HOX* genes play a role in the specification of MSC identity [45,46]. Indeed, umbilical cord MSC (UC-MSC) and bone marrow MSC (BM-MSC) show a different profile of *HOX* gene expression. *HOXA9*, *HOXB7*, *HOXC10* and *HOXD6* are expressed in UC-MSC, whereas BM-MSC express *HOXB7* and *HOXD6* [47]. 

Patterns of *HOX* gene expression can also distinguish stem cell populations of functionally distinct tissues and influence their differentiation potential. For instance, the *HOX* code of skeletal stem/progenitor cells (SSC) can affect their differentiation into resident tissue osteoblasts. These stem cells show a *HOX* gene expression profile based on their embryonic origin. Therefore, mouse SSC in the tibia maintain their embryonic identity being of mesodermal origin and expressing *Hoxa1*. On the other hand, those in the mandibular originate from the neural crest and do not express *HOX* genes. This positional memory has been shown to affect the differentiation and regeneration potential of SSC when ectopically transplanted as the original *HOX* code is retained upon transplantation [48]. A similar behavior can be observed in two populations of cord blood stem cells, the UC-MSC and the unrestricted somatic stem cells (USSC). In contrast to UC-MSC, USSC do not express *HOX* genes, and therefore they retain the potential to differentiate into three germ layers similarly to ESC [47].

*HOX* genes also play a critical role in lineage restriction, as shown during the differentiation of the hematopoietic system. In this lineage, *HOXA9*, *HOXB3,* and *HOXB4* are expressed in hematopoietic stem cells (HSC) which can differentiate into myeloid cells predominantly expressing *HOXA* genes, and erythroid and lymphoid cells expressing *HOXB* and *HOXC* genes, respectively [49,50]. *HOX* genes are also involved in the differentiation of other lineages. For example, *HOXA10* mediates osteogenic differentiation [51], whereas *Hoxa3* and *Hoxd3* induce differentiation of endothelial cells and angiogenesis in the mouse [52]. In the nervous system, 3’ *HOX* genes play a dominant role in neurogenesis and *HOXB4* can drive neural differentiation in the neural tube, whereas *Hoxb1* is essential for driving mouse neural stem cells to hindbrain [53]. Finally, several studies demonstrated a role for *HOX* genes in the regulation of terminal differentiation and tissue maturation. For instance, *HOXA5* and *HOXD10* are required for maintaining differentiated endothelial cells in a mature and quiescence state [28]. Similarly, *Hoxa2* and *Hoxb1* regulate the maturation of facial nerves in the mouse [54]. Although these studies suggest a positive effect of *HOX* gene expression during terminal differentiation, final tissue maturation can also require downregulation of *HOX* gene expression. This is the case for mature bone marrow cells that require downregulation of *HOX* genes specifying the hematopoietic lineage in order to terminally differentiate [49]. Altogether, these studies show the importance of the *HOX* code in maintaining ASC function and restriction of differentiation programs. 

## 5. *HOX* Genes in Cancer Stem Cells 

The fidelity of *HOX* gene expression in stem cells and their differentiated progenies is critical for normal tissue homeostasis. Consequently, alteration of *HOX* gene expression can play a critical role in the development of cancer [55]. *HOX* genes are frequently deregulated in cancer and many studies have shown they can function as tumor modulators by playing either an oncogenic or a tumor suppressive role [56]. Indeed, upregulation of *HOX* genes that are normally expressed in undifferentiated cells drives oncogenesis, whereas downregulation of *HOX* genes that are normally expressed in differentiated tissues results in the abolition of their function as tumor suppressors [57]. An altered expression of defined *HOX* clusters is found in different cancers, including alteration of *HOXA* genes in breast and cervical cancers, *HOXB* in colon cancer, *HOXC* in prostate and lung cancers, and *HOXD* in colon and breast cancer [58,59]. The involvement of *HOX* genes in cancer is therefore complex, with mechanisms of deregulation of specific *HOX* genes differing among cancer types [55].

The role of *HOX* genes in maintaining cell identity limits the ability of cells to transition between different phenotypes. However, research on *HOX* genes during regeneration and wound healing in lower organisms have highlighted a role for these transcription factors in dedifferentiation and in shifting cells between metastable states. Therefore, aberrant *HOX* gene expression can lead to loss of differentiation and increased cell plasticity in the context of regeneration [60].

In cancer, this type of phenotypic plasticity can drive cells to re-acquire self-renewal and a stem cell phenotype, thus leading to the formation of cancer stem cells (CSC) and tumor initiation [12]. CSC are malignant stem/progenitor cells that have been identified in many different tumor types [61,62]. They can originate from transformation of normal tissue-resident stem/progenitor cells or reprogramming of differentiated cells [63,64]. Irrespective of their origin, CSC retain a high degree of plasticity and the ability to both proliferate and give rise to the heterogeneous bulk of the tumor in response to signals from the tumor microenvironment. CSC are involved in tumor initiation, progression, invasion, resistance to treatment and are directly linked to poor clinical outcome [65,66,67]. The formation of CSC involves the acquisition of a similar epigenetic landscapes to that of normal stem cells [12], with bivalent chromatin marks and DNA methylation dynamically regulating differentiation and stemness genes [68].

Aberrant epigenetic regulation of *HOX* gene expression is common in cancer and can contribute to CSC plasticity [69]. By screening homeobox gene expression in breast CSC, we demonstrated that epigenetic silencing of *HOXC8* mediated by MIR-196 and DNA methylation induces a CSC phenotype in normal mammary stem cells, and it results in their increased self-renewal, impaired differentiation, and augmented tumorigenic potential [70]. A similar phenomenon was observed in other types of CSC. Epigenetic alteration of *HOXD9* and *HOXA10* in glioma CSC induces cell proliferation and survival [71,72]. Similarly, hypomethylation of the RA-cis regulatory element in leukemic stem cells induces expression of *HOXB* cluster genes driving the expansion of the malignant HSC pool [73]. Epigenetic regulation through expression of *HOX*-derived lncRNA can also influence the function of CSC [74]. HOTAIR expression can regulate the process of epithelial-to-mesenchymal transition (EMT) and acquisition of a CSC phenotype. HOTAIR sustains EMT induced by TGFβ in colon CSC [75], and it suppresses the tumor suppressor activity of MIR-7 via regulation of *HOXD10* whilst sustaining the expression of genes inducing EMT in breast CSC [76]. HOTAIR plays an important role in the transformation of CSC also in lung, liver, and brain cancers [77,78,79]. HOTTIP, another lncRNA derived from the *HOX* cluster, has also been shown to be involved in CSC function. In pancreatic cancer, HOTTIP enhances CSC properties by induction of *HOXA9* and activation of the Wnt pathway [80].

Further studies have shown the role of *HOX* genes in CSC transformation. For instance, *HOXA4* and *HOXA9* are enriched during the transformation of colon CSC, and their expression contributes to sustained CSC self-renewal [81]. Upregulation of *HOXA9* has also been shown to sustain the self-renewal of HSC in acute myeloid leukemia (AML) and the NUP98-HOXA9 fusion protein induces long-term proliferation and impaired differentiation of leukemic CSC [82,83]. In the breast, *HOXB3* has been shown to sustain the proliferation and drug resistance of breast CSC [84]. Finally, downregulation of *HOXA5* and RA signaling in mammary cells leads to loss of the epithelial phenotype and acquisition of CSC characteristics [85]. *HOXA5* plays a similar role in colon cancer, as silencing of *HOXA5* by Wnt signaling maintains the pool of CSC and reactivation of *HOXA5* by RA treatment induces loss of the CSC (Table 1) [86].

Overall, the mentioned studies show a clear role of *HOX* genes in CSC function; however, the functional gene networks downstream of *HOX* activation still needs to be fully determined.

The deregulation of *HOX* genes has an impact on key processes that sustain CSC function, including self-renewal, cell death evasion, and the ability to metastasize. *HOX* genes can sustain cell proliferation via autocrine stimulation by growth factors and stimulation of cell cycle progression and resistance to cell death [88]. For example, *HOXB7* activates the FGF-MAPK (fibroblast growth factor- mitogen-activated protein kinases) and PI3K/Akt (phosphoinositide 3-kinase/protein kinase B) signaling pathways in breast and gastric cancer, respectively [89,90]. Other *HOX* genes have a direct effect on cell cycle progression by inducing expression of Cyclin D1, like *HOXB7* in colorectal cancer [91] and *HOXA9* in leukemia [92]. HOX genes are also involved in the acquired resistance to cell death. Indeed, *HOXB7* can induce resistance to cytotoxic drugs [93,94], and loss of *HOXA5* results in resistance to apoptosis induced by TP53 in breast cancer [95].

Another important CSC characteristic regulated by *HOX* genes is invasion and metastasis. Several *HOX* genes are involved in the regulation of EMT, a process involved in metastatic spread and acquisition of CSC characteristics. This role has been demonstrated for *HOXB7* which promotes EMT in breast cancer [89], and for *HOXA10* whose silencing induces EMT in endometrial and lung cancer [96,97]. 

## 6. *HOX* Genes and CSC Targeted Therapies

Therapeutic targeting of CSC is a growing area of research, with drug discovery programs aimed at finding specific therapies that can eradicate this tumor initiating and disseminating cell population. Current evidence supports an important role played by *HOX* genes in the regulation of both normal and CSC function. Therefore, *HOX* genes may represent novel cancer biomarkers for targeted therapy and prediction of drug response and prognosis. Although targeting *HOX* genes represents an exciting opportunity, many challenges remain. First, transcription factors are not easily “druggable”. Targeting these proteins is difficult, mainly due to their intracellular or nuclear localization and their interaction with different cofactors [98]. This limitation could be overcome by developing targeted gene editing approaches coupled with novel drug delivery systems, and this certainly represents a new avenue with a great potential.

Another possibility would be to interfere with targets downstream of the *HOX* genes. This approach is also particularly important when considering the functional versatility of *HOX* genes. As previously discussed, the same *HOX* gene can play different roles by functioning either as an oncogene or a tumor suppressor in different cancer types. In addition, some *HOX* genes with conserved patterning function present a high degree of redundancy, and therefore the inhibition of one gene can be overcome by expression of a paralogous gene or another gene in the same cluster [99,100]. Targeting the interaction of HOX proteins with their cofactors PBS or MEIS could have the potential to inhibit the oncogenic potential of *HOX* genes in a specific cellular context. The inhibition of HOX–PBX complexes has already been achieved by using the antagonist peptide HXR9 which can induce apoptosis in a number of cancers, including solid tumors of the breast, prostate, ovarian, kidney, skin, lung, and blood tumors such as myeloma and AML [101].

Although these approaches seem promising, therapeutic targeting of CSC remains a clinical challenge, mainly due to their inherent plasticity that is influenced by the tumor microenvironment. Therefore, strategies aimed at targeting the microenvironment as well as CSC could prove effective. For example, *HOXA5* and *HOXD10* are expressed in normal epithelial and endothelial cells in the breast and their silencing is associated with CSC expansion and cell migration. Restoring the expression of both *HOX* genes could therefore be more effective and limit plasticity by targeting both CSC and angiogenesis [102,103,104].

Epigenetic therapies could also be instrumental for targeting *HOX* gene expression in CSC and affect their function by reducing self-renewal and promoting differentiation [105]. This approach is viable as several histone demethylase and histone deacetylase inhibitors are already clinically approved and many are currently being tested in clinical trials. Differentiation therapy approaches are also effective in targeting CSC by re-establishing normal *HOX* gene expression. RA can induce terminal differentiation of CSC, and it is currently used as standard of care for the treatment of acute promyelocytic leukemia [106]. We have also shown that RA is effective in re-establishing *HOXC8* expression in breast CSC [70], and other studies demonstrated the same effect in restoring expression in *HOXA5* in colon and breast CSC [85,86].

*HOX* gene expression could also inform cancer prognosis given that cancers that are enriched with CSC are also associated with a worse patient outcome. An association between *HOX* expression and poor prognosis has already been established for breast cancer and lung adenocarcinoma (*HOXB7*), renal clear cell carcinoma (*HOXC11*), mesothelioma (*HOXB4*), oral squamous cell carcinoma (*HOXD13*), gastric cancer (*HOXC6*), thyroid cancer (*HOXC10*), bladder cancer (*HOXB13*), and AML (*HOXA9*) [101]. In addition, HOX gene expression profiling in circulating CSC could provide a powerful prognostic tool for the development of personalized therapies [107]. We have shown that *HOX* gene profiles in CSC defines breast cancer molecular subtypes, suggesting that their expression could be used as a blueprint for defining the cell of origin of different types of cancer and inform clinical decisions [70].

## 7. Conclusions

**Figure 1:**
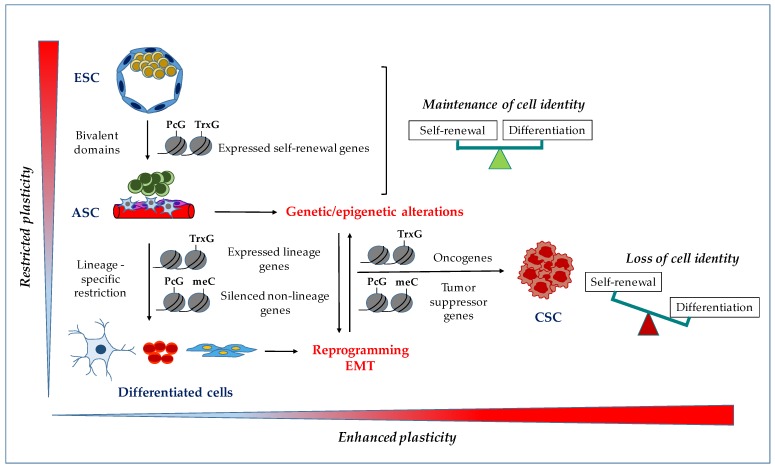
Role of *HOX* genes in the regulation of normal and cancer stem cells. *HOX* genes in ESC and ASC are epigenetically regulated by PcG and TrxG proteins. These epigenetic marks maintain stem cell identity and allow the expression of *HOX* genes in stem cells to regulate self-renewal and differential potential. After differentiation, lineage restriction is maintained by PcG proteins and DNA methylation. Genetic and epigenetic alterations induce reprogramming and EMT in either ASC or differentiated cells to induce their transformation into CSC. Re-expression of embryonic *HOX* genes and silencing of differentiation-specific genes drive CSC tumorigenicity and increase CSC plasticity. Abbreviations: ESC: embryonic stem cells; ASC: adult stem cells; PcG: Polycomb group of proteins; TrxG: Trithorax group of proteins; EMT: epithelial-to-mesenchymal transition; CSC: cancer stem cells.

*HOX* genes are considered master regulators of cell fate determination during embryonic patterning. Positional and cell-specific *HOX* identities established during development are maintained during adulthood and enable tissue homeostasis. A specific *HOX* code is concealed in adult stem cells, which allows their self-renewal and differentiation potential. This code is then resolved during differentiation into different lineages, resulting in tissue-specific expression of *HOX* genes. In cancer, erasure of the *HOX* code causes a loss of cell identity and acquisition of a transformed phenotype and CSC characteristics (Figure 1). Current evidence supports a role of *HOX* genes in the function of normal and cancer stem cells, but the molecular mechanisms by which *HOX* gene deregulation alters stem cell function are still not fully known. Therefore, large scale genomic and proteomic approaches are needed to elucidate the function of *HOX* genes and their mode of action in regulating stem cells in normal and cancerous tissues. Given the critical role of *HOX* genes in lineage specification and acquisition of cell identity, *HOX* profiles could allow the development of new diagnostic and prognostic tools for personalized clinical management of cancer patients. In addition, they could be used to devise targeted therapies that could eliminate the so far elusive CSC.

## Figures and Tables

**Table 1:** *HOX* genes and HOX-cluster derived lncRNA in CSC.

HOX Genes and lncRNA	CSC Type	Function
*HOXA5*	Breast	Silencing of *HOXA5* induces loss of differentiation [85]
	Colon	Silencing of *HOXA5* maintains the pool of CSC [86]
*HOXA4*	Colon	Expression of *HOXA4* induces CSC self-renewal [87]
*HOXA9*	AML	*HOXA9* and NUP98-HOXA9 fusion protein sustain self-renewal and impair differentiation of CSC [82,83]
	Colon	Expression of *HOXA9* induces CSC self-renewal [87]
*HOXA10*	Glioblastoma	Activation of *HOXA10* by the TrxG protein MLL induces tumorigenicity of CSC [72]
*HOXB* cluster	Leukemia	Expression of *HOXB* genes induces expansion of CSC [73]
*HOXB3*	Breast	Expression of *HOXB3* sustains proliferation of drug resistance of CSC [84]
*HOXC8*	Breast	Silencing of *HOXC8* sustains self-renewal and impairs differentiation of CSC [70]
*HOXD9*	Glioma	Silencing of *HOXD9* induces self-renewal and survival of CSC [71]
HOTAIR	Colon	Expression of HOTAIR induces EMT and stemness [75]
	Breast	Expression of HOTAIR induces EMT and stemness through activation of *HOXD10* [76]
	Lung	Expression of HOTAIR induces EMT and stemness [78]
	Liver	Expression of HOTAIR induces EMT and stemness [77]
	Glioma	Expression of HOTAIR induces proliferation and invasion of CSC [79]
HOTTIP	Pancreas	Expression of HOTTIP induces CSC proliferation by induction of *HOXA9* [80]
